# An Integrative Review of PrEP Promotion Strategies at Historically Black Colleges and Universities To Reduce HIV Disparities

**DOI:** 10.1007/s11904-025-00764-x

**Published:** 2025-11-10

**Authors:** Deja Knight, James Gilbreath, Tiara C. Willie, Jessica Corcoran

**Affiliations:** 1 Department of Epidemiology, Johns Hopkins Bloomberg School of Public Health, 624 N. Broadway, Baltimore, MD 21205 USA; 2 Lister Hill Library of the Health Sciences, The University of Alabama at Birmingham, 1701 University Blvd, Birmingham, AL USA; 3 Department of Mental Health, Johns Hopkins Bloomberg School of Public Health, 624 N. Broadway, Baltimore, MD 21205 USA; 4 Family, Community, and Health Systems Department, School of Nursing, The University of Alabama at Birmingham, 1701 University Blvd, Birmingham, AL USA

**Keywords:** PrEP, Pre-exposure prophylaxis, HBCUs, HIV, Black, United states

## Abstract

**Purpose of Review:**

We searched PubMed, CINAHL Plus, Embase, Scopus, Web of Science, and ERIC to identify peer-reviewed studies published between 2015 and 2025 that discussed PrEP at HBCUs. We sought to examine PrEP research, with a focus on PrEP implementation strategies, at HBCUs. Two authors reviewed titles, abstracts, and full texts against the inclusion criteria. Six articles were included in this review.

**Recent Findings:**

Two studies focused on formative PrEP research, two on organizational readiness, and three on post-implementation outcomes. Formative research showed PrEP is acceptable to HBCU students, though barriers like stigma and side effects, along with facilitators such as personal PrEP stories, impact intention. Organizational readiness to implement PrEP varied, with resource levels affecting implementation. Despite challenges, PrEP interventions were shown to be effective at increasing PrEP knowledge and behavioral intention.

**Summary:**

There is a need to enhance PrEP implementation at HBCUs, while considering varying campus resources.

## Introduction

In the United States (U.S.), Black Americans continue to be disproportionately impacted by HIV [1]. Although Black individuals make up approximately 12% of the population, they accounted for 38% of the 37,981 new HIV diagnoses among individuals aged 13 and older in 2022 [1]. The HIV incidence rate among Black individuals was 41.6 per 100,000, which is about eight times higher than the rate for White individuals (5.3 per 100,000) and nearly twice the rate for Latino individuals (23.4 per 100,000) [2, 3]. Black men, in particular, faced the highest HIV incidence rates among all racial and ethnic groups, with 66.3 diagnoses per 100,000 [2]. Among U.S. adolescents and young adults 15 to 24 years of age, the disparity in HIV incidence rates is even more pronounced with Black adolescents and young adults accounting for more than half of all new HIV diagnoses among 15- to 24-year-olds [1–3]. These HIV incidence disparities highlight the urgent need for targeted HIV prevention efforts within Black communities and more specifically for Black adolescents and young adults.

Antiretroviral medication for pre-exposure prophylaxis (PrEP) is more than 90% effective when used consistently for HIV prevention, but PrEP is underutilized by adolescents and young adults [4–8]. In 2022, according to AIDSvu, an interactive online mapping tool that visualizes the impact of the HIV epidemic on communities across the U.S., the adolescent and young adult population was highlighted as the age group with the greatest unmet need for PrEP with only 12% of PrEP users being between the ages of 13 to 24 years of age [9]. Additionally, there is an unmet need for PrEP among Black communities [10]. When examining racial differences in PrEP uptake in 2022, only 14% of PrEP users were Black; yet they comprised 38% of new HIV incident cases [9]. Due to the high HIV incidence rates among Black adolescents and young adults, the slow uptake of PrEP could further exacerbate racial disparities in HIV incidence [5, 9].

Historically Black College and University (HBCU) campuses are priority locations to pilot interventions aimed at increasing PrEP access for several reasons including that HBCUs are viewed as places of influence among the community and Black young adult leaders are cultivated within HBCU environments [11–13]. In addition to increasing PrEP access, understanding the factors that make PrEP accessible and acceptable to HBCU student populations is integral to engaging students. Unfortunately, there is insufficient literature currently published on PrEP at HBCUs.

There is a need for culturally tailored sexual health education and intervention programs that address the unique experiences and challenges faced by HBCU students. A 2013 review of sexual health behaviors and risk factors among students at HBCUs found that there was a disconnect between perceived risk and actual behavior, as many students underestimated their vulnerability for STIs and HIV, leading to inconsistent condom use and multiple sexual partners. Despite possessing awareness of safe sexual practices, many students engaged in unsafe sexual behaviors. Gaps in comprehensive sexual education persisted, resulting in misinformation and a lack of preventative actions. To improve sexual health outcomes at HBCUs the study concluded that community and peer support, as well as collaborative work with HBCU populations, is needed [14]. Recent work at an HBCU examining STI testing, HIV testing, and sexual behaviors echoed the call for continued work examining testing and best practices to reach college students attending HBCUs for HIV prevention [15].

As of 2022, there were 99 HBCUs across the U.S. with a total enrollment of almost 300,000 students [16]. With advances in PrEP access and PrEP delivery methods, reviewing the recent literature around PrEP at HBCUs is critical to move this area of science forward to ultimately improve sexual health outcomes for HBCU students who could benefit from targeted HIV prevention efforts.

## Methods

The literature search for this integrative review was conducted by a research librarian. The databases that were used for this search were: PubMed, CINAHL Plus with Full Text, Embase, Scopus, Web of Science, and ERIC. The search consisted of terms related to HBCUs, including a full list of HBCU institutions as classified by the US Department of Education (except PubMed, which utilizes a phrase index), and terms related to PrEP [17]. The only search restriction was the date range of January 1st, 2013, to the date of the search execution, February 23rd, 2025. For the purposes of deduplication and screening, the researchers utilized Covidence [18].

After removing 99 duplicates using Covidence, 110 studies remained for screening. Two authors (D.K. and J.C.) independently reviewed titles and abstracts for relevance, applying predefined inclusion and exclusion criteria. Studies were included if they [1] were written in English [2], presented original, empirical, peer-reviewed data [3], were conducted in the United States [4], were conducted at a HBCU, and [5] discussed PrEP and/or a HIV prevention intervention. Articles were excluded if they were review articles, opinion pieces, conference abstracts, or only discussed HIV risk behaviors. After the title and abstract review, 32 articles underwent full text review. Our full text review resulted in the exclusion of 26 studies due to reasons including incorrect outcomes (i.e., did not focus on PrEP or a HIV intervention), non-peer-reviewed status (i.e., dissertations), wrong patient population (i.e., research not conducted at an HBCU) or inability to access the full text. Ultimately, six studies were included in this integrative review.

**Fig. 1:**
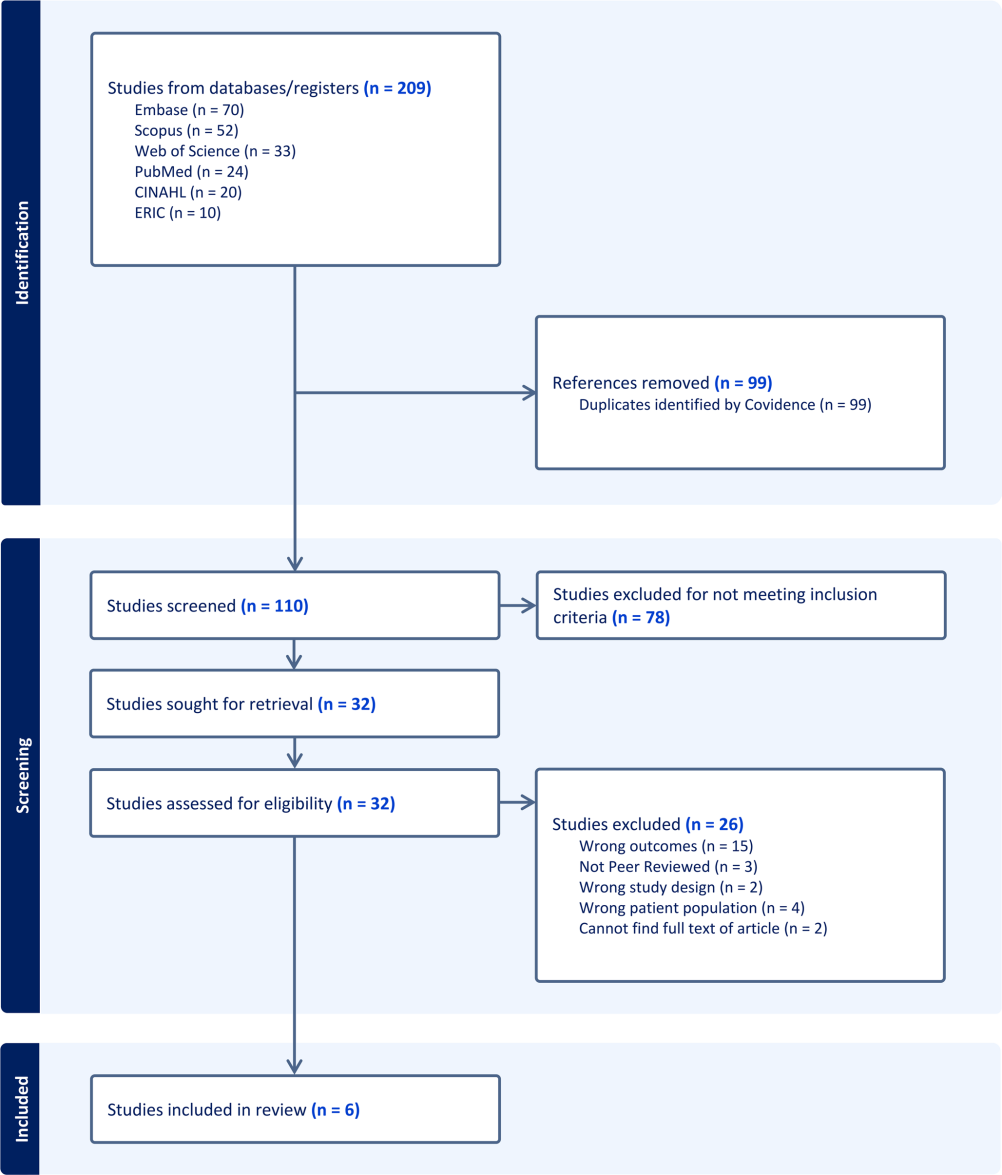
HBCU and PrEP / HIV / STI testing review

For the six eligible articles, we extracted the following information into an Excel database: Title, authors, year of data collection, geographic location, study type (qualitative, quantitative, or mixed methods), and key findings related to PrEP. Based on the key findings, we grouped each article into one of three categories: formative research, pre-implementation, or post-implementation of an HIV and/or PrEP intervention. The extracted data were, then, synthesized within each group, with themes presented according to the aims of this scoping review. Discrepancies between reviewers were resolved through weekly discussions until consensus was reached. A PRISMA diagram with the complete literature review process is included in Fig. 1.

## Results

Overall, six articles were included in this integrative review. Table 1 includes each article title, year of data collection, geographic location, study design, and key findings. Of the six articles, three were quantitative (all using surveys), two were qualitative (using a combination of individual interviews and focus groups) and one utilized a mixed methods design (both qualitative interviews, focus groups, and a survey). Studies were conducted in various places like the Southeast, Northeast, as well as North Carolina, Florida, and Georgia. The studies spanned the full implementation research continuum. Formative research focused on uptake, including awareness, acceptability, barriers and facilitators of PrEP. Pre-implementation research highlighted including the benefits of collaborating with community organizations to increase knowledge and access to PrEP. Post-implementation research evaluated interventions to increase PrEP delivery to HBCU students and to strengthen healthcare providers’ knowledge and confidence in prescribing PrEP.

**Table 1:** Descriptions of studies published on HIV prevention interventions and PrEP at hbcus

Title	Authors	Year of Data Collection	Geographic Location	Study Type (Qual, Quant, MM)	Key Findings Related to PrEP and HIV prevention
Implementation of an HIV Prevention Intervention at Historically Black Colleges and Universities and Predominantly Black Institutions	Holliday, R. C.; Martin, S. D.; Phillips, R.; Shahin, Z.; Farley, K.; Cahoy, A. B.; Ross, T.	2020–2021 and 2024	Three HBCUs and a predominately Black institution in a southeastern state	Qualitative	Barriers and facilitators to implementing an HIV prevention intervention on four campuses highlight several key issues. The need for the intervention is clear, and it could be implemented without major difficulty, although some campuses lack a dedicated student health center. Despite limited resources and personnel, key informants believe the intervention can still proceed, with external partnerships potentially providing funding for HIV testing. Cost concerns remain a challenge, and the institutional culture of religious schools complicates open discussions and education about HIV. Access to health resources on campus is preferred due to transportation limitations, but students often don’t see HIV prevention as a priority or may not feel at risk. Stigma around testing and the focus of resources on pregnancy prevention are additional obstacles, while HIV testing is currently only available twice a week. Post-implementation barriers and facilitators to an HIV prevention intervention include the institutional culture, which creates divisions among students and staff regarding discussions on sexual health and HIV. External partnerships helped by providing staff and resources to support the intervention, and increased access to resources led many to view the intervention as successful. However, internal communication issues hindered marketing and uptake, and challenges in recruiting and retaining peer educators arose due to stigma and conflicting priorities.
Increasing provider awareness of PrEP on HBCU campuses and beyond: A case study of the HBCU HIV prevention project (H2P)	Downer, G. A.; Cunningham, S. R.; Ramsey, L. M.; Ellick, K. L.; Bailey, D.	Not specified	HBCU in an unspecified location	Quantitative	The study highlighted three key takeaways: First, the H2P intervention successfully enhanced provider knowledge and awareness of PrEP and HIV prevention, with off-campus providers showing greater readiness to apply the training in practice. Second, the impact of the intervention was sustained over time, as providers continued to apply the knowledge gained, particularly off-campus providers, who demonstrated strong daily use of PrEP-related knowledge. Lastly, while on-campus providers showed knowledge gains, they were less likely to consistently apply the training or feel confident discussing PrEP, underscoring the need for ongoing support, technical assistance, and further training to ensure sustained effectiveness in PrEP promotion on HBCU campuses.
The HIP LADIES: A Pilot Health Improvement Project for HIV Prevention in Black College Women	Chandler, R.; Ross, H.; Paul, S.; Shittu, A.; Lescano, C.; Hernandez, N.; Morrison-Beedy, D.	January 2014 - October 2014	Two HBCUs in Florida	Quantitative	The HIP LADIES intervention was both feasible and well-received, with participants expressing that it was helpful and acceptable. The evaluation showed significant improvements in HIV knowledge, future optimism, and behavioral intentions in the intervention group compared to the control group. While social norms and condom attitudes were higher in the intervention group, they were not statistically significant. There were no differences between groups in condom use, sexual assertiveness, or sexual experience. Overall, the intervention notably enhanced HIV knowledge and appeared appropriate for the target audience, emphasizing the importance of considering the social context of HIV risk behaviors and women’s future aspirations.
Navigating Pre-exposure Prophylaxis Access: Qualitative Insights From Black Women at a Northeastern Historically Black College and University	Robinson, M.; Aidoo-Frimpong, G.; Nelson, L.; Sandoval-Rosario, M.; Williams, B.; Chandler, R.	Spring 2022	HBCU located in the Northeastern region of the United States	Qualitative	The study on PrEP and HIV prevention among Black women at a Northeastern HBCU identified key barriers and facilitators to PrEP adoption. Barriers included stigma, where participants feared being perceived as HIV-positive due to societal misconceptions; concerns about side effects, including potential long-term health and fertility impacts; and financial obstacles, particularly regarding insurance and affordability. On the other hand, facilitators included the effectiveness of PrEP in preventing HIV, a perceived high risk of exposure, and engaging in unprotected sex, even within committed relationships. The study also highlighted the need for culturally relevant education and outreach to increase PrEP uptake, with recommendations for real-life testimonials, social media engagement, and endorsements from trusted community figures. These findings stress the importance of addressing stigma, improving accessibility, and tailoring awareness campaigns to encourage PrEP use among Black women at HBCUs.
Awareness and acceptability of HIV pre-exposure prophylaxis (PrEP) among students at two historically Black universities (HBCU): a cross-sectional survey	Okeke, N. L.; McLaurin, T.; Gilliam-Phillips, R.; Wagner, D. H.; Barnwell, V. J.; Johnson, Y. M.; James, O.; Webb, P. B.; Parker, S. D.; Hill, B.; McKellar, M. S.; Mitchell, J. T.	February 2018 - April 2018	Two HBCUs in North Carolina	Quantitative	PrEP awareness among HBCU students is growing, with 52% of students familiar with it, though many learned about it recently—61% within the past six months. Despite this increase in awareness, 73% of students perceived themselves as “not at risk” for HIV, with only 9% considering themselves at moderate or high risk. However, 69% expressed interest in using some form of PrEP, indicating a disconnect between students’ risk perceptions and their willingness to adopt PrEP. Campus health services were the primary sources of PrEP information, highlighting their key role in spreading awareness. These findings suggest a need for targeted educational efforts to address the gap between students’ HIV risk perceptions and PrEP use, while leveraging campus resources to improve outreach and access.
Awareness, knowledge, and willingness to take PrEP among Black students attending Historically Black Colleges and Universities and Minority Serving Institutions	Holliday, R.; Martin, S.; Phillips, R.; Barrett, T.; Shahin, Z.; Farley, K.; Bunzy, N.; Kegley, S.	2020–2021	Three HBCUs and two Minority Serving Institutions in Georgia	Mixed Methods	Awareness and understanding of PrEP among students were limited, with only 44% having heard of it, and many expressing confusion about its purpose and how to access it. Misconceptions, such as believing it was only for gay men, highlight the need for targeted educational efforts. Despite over 60% of students being sexually active and nearly 48% engaging in unprotected sex, many did not perceive themselves at significant risk for HIV, potentially contributing to low PrEP uptake. Barriers to access included cost, limited on-campus health services, and inconsistent partnerships with external providers, with only one institution offering direct access to PrEP, forcing students to seek services elsewhere, raising privacy and logistical concerns.

**Fig. 2:**
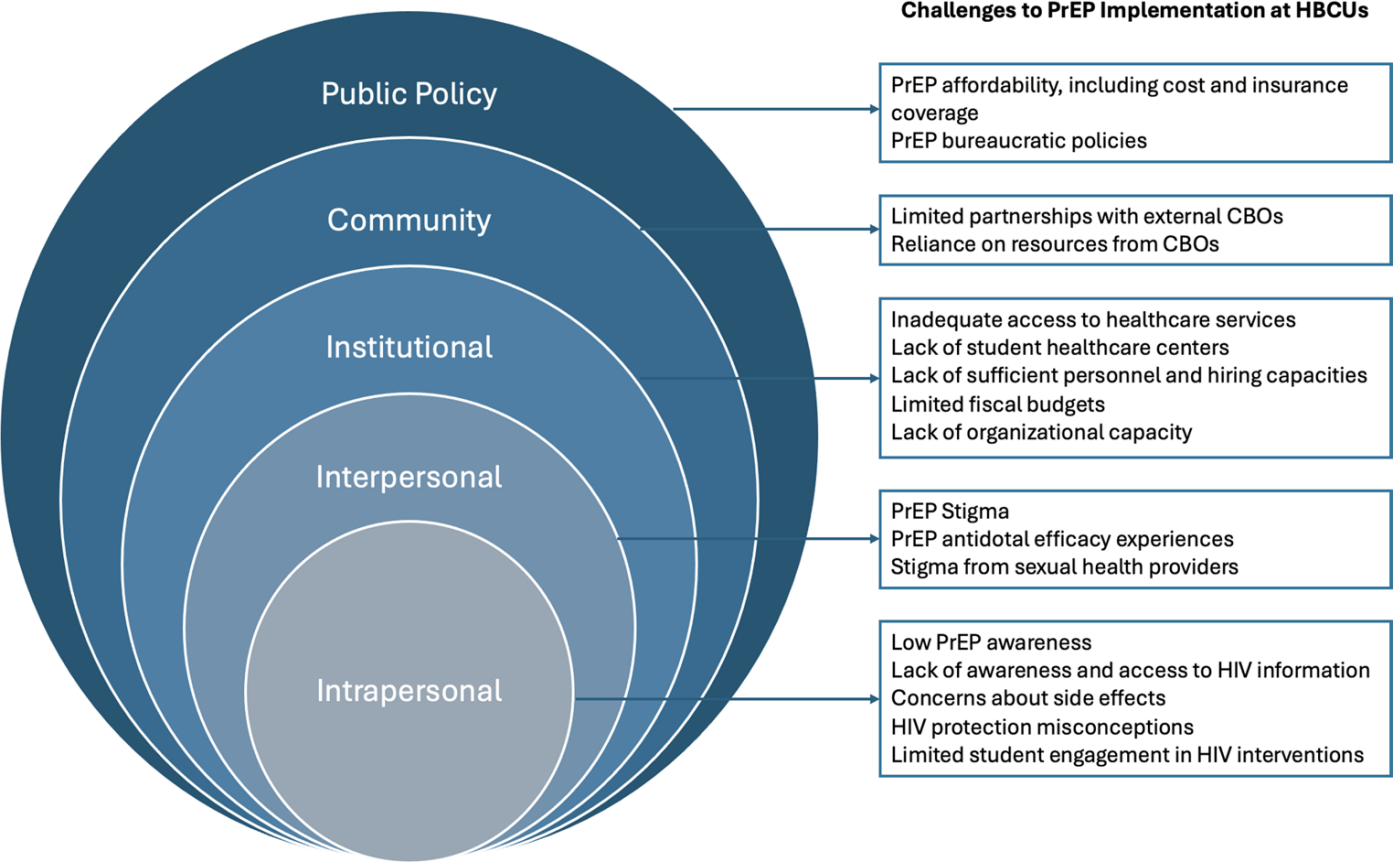
Factors influencing PrEP implementation on the campuses of HBCUs in the United States

Figure 2 depicts a synthesis of current knowledge and remaining research gaps related to PrEP implementation at HBCUs, organized using the socio-ecological framework [19]. In this version of the model, health behavior is viewed as being determined by multiple levels of influence, including intrapersonal factors (such as individual knowledge, attitudes, and skills), interpersonal relationships (such as social networks and support systems), institutional factors (such as organizational rules and structures), community factors (such as relationships among organizations), and public policy (such as local, state, and national laws) [19].

### Formative Research on PrEP Uptake [20, 21]

Two studies discussed formative research on PrEP uptake including findings on (a) PrEP awareness and acceptability, (b) barriers to PrEP uptake, and (c) facilitators to PrEP uptake. Okeke et al. (2021) administered a cross-sectional survey to students at two HBCUs in North Carolina to assess HIV risk perception, PrEP awareness, and PrEP acceptability [20]. Robinson et al. (2024) conducted focus groups with 22 Black HBCU women in the northeastern region of the United States to identify barriers and facilitators to PrEP uptake [21]. The following sub-sections presents findings from these two articles.

#### PrEP Awareness and Acceptability [20]

Awareness and acceptability of pre-exposure prophylaxis (PrEP) among participants indicate promising opportunities for broader adoption of this HIV prevention method [20]. Approximately 50% of participants reported awareness of PrEP, with the majority learning about it within the past three months [20]. A quarter of participants indicated that they first encountered PrEP information through the student health center, while fewer than 25% were introduced to it via health promotion events organized by student health services [20]. More than half of the participants expressed willingness to use either a daily oral pill or a 2-month injectable formulation of PrEP [20]. Notably, around 50% also stated they would be open to a monthly injectable option [20]. These findings suggest that a significant proportion of participants find at least one form of PrEP acceptable, highlighting potential avenues for broader adoption and access among this population [20].

#### Barriers To PrEP Uptake [21]

Black college women face significant barriers to PrEP uptake, including stigma, concerns about side effects, and financial challenges. Stigma, in particular, was a prominent factor, as many participants expressed apprehension about the negative perceptions associated with PrEP use [21]. Specifically, they were concerned that using PrEP could lead to being misidentified as HIV-positive, reinforcing stereotypes that HIV predominantly affects certain communities [21]. Additionally, concerns about potential side effects of PrEP, including their severity, deterred some Black women from considering it as a viable option [21]. Financial barriers were also a major concern, with participants highlighting issues related to the long-term affordability of PrEP, insurance coverage, and overall cost [21]. These multifaceted barriers—stigma, health concerns, and financial strain—collectively influenced Black college women’s decisions regarding PrEP, underscoring the need for tailored interventions to address these challenges and promote wider acceptance and access [21].

#### Facilitators to PrEP Uptake [21]

Personal experiences and perceptions of HIV risk significantly influence Black college women’s decisions regarding PrEP use. The effectiveness of pre-exposure prophylaxis (PrEP), as demonstrated through anecdotal evidence, played a pivotal role in motivating Black college women to consider using PrEP [21]. This suggests that personal narratives and shared experiences are crucial decision-making factors for this population [21]. Additionally, perceptions of HIV exposure or risk of exposure significantly influenced their decisions regarding PrEP [21]. Participants also acknowledged the connection between unprotected sex and both increased HIV risk and the potential for prevention through PrEP [21]. However, an interesting finding emerged in relation to contraceptive use: many Black college women underestimated the importance of protecting themselves from HIV, believing that birth control alone offered sufficient protection [21]. This highlights a potential gap in knowledge regarding the distinct roles of contraception and HIV prevention methods, underscoring the need for targeted education on comprehensive sexual health protection [21].

### Organizational Readiness To Implement PrEP at HBCUs [22, 23]

Two studies discussed organizational readiness to implement PrEP at HBCUs including findings on: (a) HBCU resources for HIV prevention initiatives and PrEP, (b) external partnerships supporting HIV and PrEP initiatives, and (c) student needs and university culture. Holliday et al. (2024a) utilized mixed methods – interviews, focus groups, and surveys – at three HBCUs and one “Minority Serving Institution” in Georgia to explore exploring PrEP acceptances, access, and services currently offered [22]. Furthermore, this study examined perceptions and experiences of HBCU students with implementing a HIV prevention intervention. Similarly, Holliday et al. (2024b) used interviews and focus groups to identify barriers and facilitators pre-implementation and post-implementation of a HIV prevention intervention. The following sub-sections presents findings from these two articles [23].

#### HBCU Resources for HIV Prevention Initiatives and PrEP [22, 23]

Participating institutions varied widely in their resources and capacity to implement PrEP services, which significantly influenced the success and sustainability of HIV prevention efforts. Many schools lacked essential resources, such as dedicated student health centers and sufficient personnel to support HIV prevention programs, including PrEP education and distribution [22, 23]. Some institutions, for instance, reported operating with reduced staff, making it challenging to establish comprehensive HIV prevention initiatives [23]. Despite these challenges, there was general institutional support for PrEP as an HIV prevention strategy, with many institutions noting that the intervention could be effectively integrated into their existing workflows if sufficient resources were available [23]. A key barrier to implementation, however, was cost, with rising insurance premiums for PrEP, limited institutional budgets, and the expense of hiring additional personnel all contributing to difficulties in sustaining these programs [22]. Lack of organizational capacity to scale up PrEP delivery, due to factors such as financial constraints, prevented students from accessing PrEP [22]. To address these challenges, some institutions explored strategies such as partnering with external organizations that provide PrEP at little or no cost or integrating the HIV prevention program with existing health initiatives to maximize available fiscal and human resources [22, 23].

#### External Partnerships Supporting HIV and PrEP Initiatives [22]

Institutions often relied on community-based organizations (CBOs) to sustain HIV prevention and education efforts, as these external partners were seen as trustworthy and reliable by students [23]. CBOs typically provided HIV testing and education, and, in many cases, also supplied condoms on campus through donations and external funding [23]. However, despite the support from these partners, few institutions had formal partnerships to support PrEP or other HIV prevention strategies [22]. Among the limited number of organizations offering PrEP education and access, partnerships were often informal and lacked structured systems for referrals and patient follow-up [22]. This inconsistency in collaboration, coupled with bureaucratic policies and administrative changes, created significant challenges in delivering continuous and reliable services to students [22]. Although institutions acknowledged these challenges, there was strong support for expanding partnerships to strengthen HIV prevention and PrEP initiatives on campuses [22].

#### Student Needs and University Culture [22, 23]

A significant barrier to PrEP uptake among students is the lack of awareness and insufficient access to HIV prevention information. Despite brief mentions of PrEP during new student orientation, students reported that the information provided was neither comprehensive nor frequent enough to counteract misinformation circulating among peers [22, 23]. Moreover, open discussions about HIV and sexual health were notably absent from campus communities, further hindering students’ understanding of these topics. To access PrEP and HIV prevention information, students had to proactively request it, rather than having it readily available from their institution [22]. Additionally, students seeking sexual health services, such as STI testing, often encountered stigmatizing attitudes from healthcare providers, with conversations focusing more on pregnancy prevention rather than broader sexual health [23]. These perceptions of stigma also extended beyond healthcare providers, as some students expressed reluctance to participate in HIV testing and condom distribution programs due to fear of judgment from their peers [23]. However, not all students shared these views; some reported that faculty and staff were supportive and nonjudgmental regarding HIV testing and sexual health services [23].

Inadequate access to healthcare services and logistical challenges contribute to students’ limited engagement with HIV prevention programs, highlighting the need for more accessible and comprehensive interventions. Many students relied on campus sexual health services, yet institutions with limited resources often required students to seek external healthcare. External referrals, however, posed several challenges, including privacy concerns and lack of follow-up, which led to poor adherence to PrEP and HIV treatment [22]. Despite these barriers, students expressed a strong desire for expanded HIV prevention programming on campus. They specifically requested increased HIV education, easier access to condoms, and more opportunities for HIV testing. Nevertheless, some students noted that academic and social commitments often competed with their ability to participate in these initiatives, indicating that programming should be designed to accommodate students’ busy schedules [23]. These findings underscore the importance of creating HIV prevention programs that are more accessible, flexible, and tailored to the needs of the student population [23].

### Findings from HIV Prevention Interventions on HBCU Campuses [23–25]

Despite persistent barriers to HIV prevention and PrEP implementation on HBCU campuses, three projects sought to address these challenges by leveraging peer support, engaging campus health providers, and forming external partnerships.

HIP LADIES used a prospective randomized two-group design to assess a structured HIV prevention program tailored for Black women at HBCUs (HIP LADIES), comparing it to a control group that received a similar educational health promotion program (C.O.S.T.). Six intervention peer facilitators were trained to deliver the intervention. Participants were recruited through a combination of electronic flyers and in-person recruiting on campus. Participants in the HIP LADIES intervention reported that the sessions were both helpful and enjoyable, with high levels of acceptance [25]. The intervention resulted in a notable increase in HIV knowledge and behavioral intentions among participants [25]. While not statistically significant, there were also higher mean scores for social norms and attitudes toward condoms in the intervention group compared to the control group [25].

The “Take CHARGE” initiative was a structured effort focused on applying three evidence-backed strategies – HIV testing, education, and condom distribution – to address HIV prevention among young Black adults at three HBCUs and one Minority Service Institution. The main goal of the project was to design and implement interventions that were culturally relevant to the norms of Black college students, incorporating community-engagement methods. Campus liaisons with assistance from community partners were used to manage implementation activities. The program placed a strong emphasis on peer involvement to provide educational materials, improve access to HIV testing, and distribute condoms. Although the initiative received support from both students and administrators, there was still some debate about whether sexual health and HIV prevention should be openly discussed on campuses, reflecting the widespread stigma surrounding HIV [23]. External collaborations with community-based organizations (CBOs) and local public health agencies, including staff support, played a crucial role in the intervention’s success. These partnerships facilitated condom distribution, peer health educator training, increased HIV testing, alleviated pressure on campus personnel, and provided technical support throughout the initiative [23]. The intervention was effective in allowing peer educators to address HIV-related misconceptions among students [23]. However, challenges such as poor internal communication on campus and difficulties in recruiting and retaining peer educators were reported as obstacles to full implementation [23].

The “HBCU-HIV Prevention Project (H2P)” is a culturally adapted intervention aimed at training healthcare providers at Historically Black Colleges and Universities (HBCUs) to play a pivotal role in reducing HIV infections and enhancing healthcare outcomes for HBCU students [24]. For this project, both on-campus healthcare providers and off-campus healthcare providers who regularly interacted with students attending nearby HBCUs were recruited. The healthcare providers participated in 11 training sessions as well as a pre and 30-day, and 60-day posttest. The intervention led to significant positive changes in the knowledge of off-campus healthcare providers, with improvements ranging from 14% to 24% [24]. These providers reported feeling more capable of applying the training’s knowledge and strategies in their daily practice [24]. Additionally, they believed that H2P helped enhance their clinical skills and better equipped them to discuss PrEP with patients [24]. On-campus providers also experienced substantial improvements in their knowledge and clinical care following their participation in H2P [24]. These positive changes were sustained at both 30 and 60 days after the intervention [24]. The results of the program demonstrated H2P’s effectiveness in increasing HIV-related knowledge among both off-campus and on-campus providers [24]. Interestingly, off-campus providers were more likely to apply the knowledge and skills gained from H2P in their practice compared to their on-campus peers [24].

## Discussion

This manuscript aimed to provide an integrative review of PrEP formative research and PrEP promotion strategies at HBCUs. After employing our search strategy, a total of six studies met our inclusion criteria and were included in this review. Of the six articles, two presented findings on formative PrEP research at HBCUs, two presented findings on pre-implementation research on PrEP at HBCUs, and three presented findings on post-implementation outcomes of PrEP implementation interventions at HBCUs. Formative PrEP research found PrEP to be acceptable to HBCU students, with notable barriers (e.g., stigma and side effects) and facilitators (e.g., personal PrEP narratives) to PrEP intention. Though HBCUs were in support of HIV prevention and PrEP interventions, pre-implementation research highlighted significant limitations in key resources vital for implementation. HBCUs reported limitations in resources such as personnel, budgets, student health centers, student engagement, and access to PrEP information. Limited opportunities for external partnerships, though these partnerships provided key resources to fulfil these deficits, were also reported. Despite persistent barriers to HIV prevention and PrEP implementation on HBCU campuses, three studies addressed these challenges by leveraging peer support, engaging campus health providers, and forming external partnerships to deliver tailored education, increase access to prevention resources, and shift social norms around HIV prevention. These findings highlight the need to expand PrEP implementation while identifying adaptable intervention strategies that can be sustained across HBCU campuses with limited resources.

We found that HBCUs had varying levels of resources that could be used to implement PrEP. One resource in particular – healthcare centers – varied greatly in access, with some HBCUs having their own center, others having limited centers, and one moving towards a combined center with another university. Though students discussed relying on healthcare centers to meet their healthcare needs, especially in the absence of reliable transportation, healthcare centers were not always available. Though majority (65%) of HBCUs were shown to have on-campus comprehensive health services with prescribing providers, a fraction of HBCUs (13%) offered no student health services [26]. Comprehensive health services were provided, in part, due to a combination of federal funding, donors, and endowments [26]. However, HBCUs are largely underfunded and rely on tuition for revenue [27]. As student health services are key for PrEP prescribing, uptake, and adherence on HBCU campuses, addressing shortages in student health care services on campuses is vital to support PrEP implementation efforts.

Limited financial resources posed a particular strain on HBCUs and PrEP implementation. HBCUs are known to function with limited fiscal resources on a daily basis, with pronounced funding inequities between HBCUs and their pre-dominantly white counterpart universities [28, 29]. The pandemic compounded these financial challenges, leaving HBCUs financially vulnerable and, at times, unable to meet the needs of their students [28]. A survey of 5,000 students who attended public and private HBCUs found that approximately two-third of students experienced basic needs insecurity [28]. HBCU students experienced insecurities in food, housing, and healthcare [28]. When HBCUs struggle to meet the basic needs of their students, it could be even more difficult to introduce PrEP on the campus. Limited fiscal resources make it difficult to effectively hire and pay personnel and to purchase supplies needed to support PrEP implementation, as highlighted in this review. Partnerships with external CBOs may provide financial and programmatic support for HIV prevention and PrEP interventions, as demonstrated in the Take CHARGE initiative [23]. However, external barriers, such as challenges with internal communication, still impacted the success of the intervention, indicating that external partnerships alone do not guarantee effective implementation. Future PrEP strategies should focus on leveraging existing campus resources while cultivating stable and long-term external partnerships to support the sustained delivery of PrEP services on HBCU campuses.

When examining organizational readiness, students expressed mixed opinions about the level of support that they feel when seeking HIV prevention services. While some students experienced stigma from healthcare staff, faculty, and administration when seeking services, others experienced full support. Stigma in HIV spaces can lead to sustained health disparities and gaps in clinical care [30]. There is a need to address and disrupt stigma against HIV prevention services in order to effectively implement PrEP [31, 32]. Two interventions sought to address student and campus cultural barriers, as well as stigma experienced from providers, to improve HIV prevention knowledge and behavioral intentions, and demonstrated promising results in shifting social norms and increasing HIV-related knowledge. Lessons learned from these interventions can inform the development of culturally responsive and stigma-reducing strategies for future PrEP implementation efforts on HBCU campuses. Future research on PrEP implementation should examine PrEP implementation in conservative spaces in order to identify strategies that may be useful in implementing PrEP on HBCU campuses that may be more conservative in nature. Additionally, future research should examine the effect of implementing cultural and bias training on HBCU campuses for healthcare personnel, faculty, and staff. Such training is necessary and valuable in increasing capacity to implement PrEP on campuses.

There are limitations that should be noted. Though we restricted our search to HBCUs, many articles did not specify whether the included institution was a HBCU. Therefore, we had to review each article to identify the institution mentioned to manually review whether it is considered a HBCU. Additionally, despite employing an extensive search strategy to find any article related to PrEP and HBCUs within the past ten years, our search strategy, including key words, could have missed some articles. Secondly, during the time of writing this review, new articles on PrEP at HBCUs could have been published, which may have also been missed. Lastly, some articles focusing on HBCU campuses solely mentioned HIV testing or risk. Though these behaviors and modalities could be related to PrEP, we excluded any article that did not directly mention PrEP or a HIV prevention intervention.

## Conclusion

This is one of the first reviews focusing on PrEP implementation strategies on HBCU campuses. Overall, we found that PrEP implementation interventions show promising positive benefits. Yet, the small number of articles in this study, including only three focusing on post-implementation outcomes, demonstrate a significant need for more comprehensive PrEP implementation strategies for HBCUs. Specifically, there is a need for more PrEP education, promotion, and access for HBCU students. These results provide insight into contributing factors for low PrEP uptake on HBCU campuses, which houses predominately Black populations.

## Data Availability

No datasets were generated or analysed during the current study.
